# Fertility preservation in women with ovarian cancer: Finding new pathways: A case-control study

**DOI:** 10.18502/ijrm.v19i2.8474

**Published:** 2021-02-21

**Authors:** Ali Khodadadian, Yasser Varghaiyan, Emad Babakhanzadeh, Iraj Alipourfard, Saeed Haghi-Daredeh, Amin Ghobadi, Mohsen Hemmati-Dinarvand, Mehrdad Talebi, Nasrin Ghasemi

**Affiliations:** ^1^Department of Medical Genetics, Faculty of Medicine, Shahid Sadoughi University of Medical Sciences, Yazd, Iran.; ^2^Department of Immunology, Faculty of Medicine, Shahid Sadoughi University of Medical Sciences, Yazd, Iran.; ^3^Center of Pharmaceutical Sciences, Faculty of Life Sciences, University of Vienna, Vienna, Austria.; ^4^School of Pharmacy, Faculty of Sciences, University of Rome Tor Vergata, Rome, Italy.; ^5^Department of Medical Nanotechnology, School of Medicine, Shahroud University of Medical Sciences, Shahroud, Iran.; ^6^Department of Clinical Biochemistry and Laboratory Medicine, Faculty of Medicine, Mazandaran University of Medical Sciences, Sari, Iran.; ^7^Department of Clinical Biochemistry and Laboratory Medicine, Faculty of Medicine, Shiraz University of Medical Sciences, Shiraz, Iran.; ^8^Abortion Research Centre, Yazd Reproductive Sciences Institute, Shahid Sadoughi University of Medical Sciences, Yazd, Iran.

**Keywords:** Ovarian cancer, Female infertility, LGR5, FOXO1, miR-340.

## Abstract

**Background:**

Surgery and chemotherapy are the two most common treatments for cancers, including ovarian cancer. Although most ovarian cancers occur over the age of 45 yr, it may involve younger women and affect their reproductive ability.

**Objective:**

To assess the expression of Leucine-rich repeat-containing G-protein coupled receptor 5 (*LGR5*), Forkhead Box O1 (*FOXO1*), and *miR-340* genes in the ovarian cancer tissues as well as ovarian cancer cell lines.

**Materials and Methods:**

In this case-control study, 30 ovarian cancer samples (with the average age of 37 ± 2.5 years) coupled with their non-tumor marginal tissue (as a control) were collected. Proliferated cell lines were treated with several concentrations of cisplatin, and the half maximal inhibitory concentration (IC50) of cisplatin was quantified by MTT-assay. After RNA extraction, cDNA synthesis and qRT-PCR were done. Finally, the results were analyzed.

**Results:**

While the expression levels of *miR-340* and *FOXO1* genes in tumor samples displayed a significant reduction (p ≤ 0.001), the *LGR5* gene presented a significant increase in expression (p ≤ 0.0001). However, conversely, the expression levels of *miR-340* and *FOXO1* genes in cisplatin-sensitive cell lines, after 24, 48, and 72 hr of cisplatin treatment, indicated a significant increase (p ≤ 0.001) while the expression of *LGR5* gene showed a significant decrease in the cisplatin-sensitive cell line (p < 0.05).

**Conclusion:**

The *LGR5*, *FOXO1*, and *miR-340* genes can be targeted for early diagnosis and more accurate treatment of ovarian cancer and may prevent some of the ovarian cancer complications such as infertility.

## 1. Introduction

Ovarian malignancies are the most common type of gynecological cancers and are often diagnosed at a later stage, when the cancer cells are migrating and invading other tissues and organs (1). Today, the standard treatments for cancer patients are chemotherapy and surgery, which in most cases lead to cure (>70% in cases of ovarian cancer); however, 90% of cases involving tumour recurrence remain incurable (2). Moreover, both methods have complications. One of the most pertinent challenges in ovarian cancer is fertility issues in women who undergo prolonged chemotherapy (especially when drug resistance is observed) or surgery (oophorectomy) (3). Oophorectomy can be bilateral or unilateral. When it is bilateral, a successful pregnancy will be impossible, and if it is unilateral, the reproductive ability will be reduced.

The effect of chemotherapy on male and female infertility has been investigated (4); that have shown a negative effect of chemotherapy drugs (including cisplatin) on fertility (5). Therefore, acquiring knowledge on all pathways involved in the formation, metastasis, growth, and drug resistance of cancer, such as ovarian cancer, can lead to the development of methods for early diagnosis and safer treatment of the disease- methods that will not require surgery and long-term prescription of chemotherapy drugs. These methods will not only reduce cancer symptoms but also minimize the complications in treated patients (e.g. infertility in ovarian cancer).

One of the most important and well-known pathways involved in the epithelial-to-mesenchymal transaction is the Wnt/β-catenin signaling pathway. The role of this pathway and its genes in many malignancies of ovarian cancer has been investigated (6). The leucine-rich repeat containing G protein-coupled receptor 5 (*LGR5*) is one of the most important genes in this pathway. The correlation between *LGR5* and aggressiveness process has been studied in previous works and the results have proved the role of the gene in this process (7). The *FOXO1* gene belongs to the forkhead transcription factors family. The role of this gene in a variety of malignancies has been examined in previous studies (8, 9). In addition, Choi and colleagues have studied the relationship between this gene and the *LGR5* gene in gastric cancer cells (10). Moreover, the role of *LGR5* and *FOXO1* gene has been addressed in some cases of female infertility who suffered from gynecological malignancies (11-14).

Micro-RNAs are non-coding regulatory small RNAs, about 17-25 nucleotides in length (15), that act as important factor in regulating the expression of genes in many biological processes; for instance, cell proliferation, cell differentiation, and death of the cell (16, 17). Through bioinformatics analysis and based on a previous study, we conclude that the *LGR5* and *FOXO1* genes can be potential targets for the *miR-340* (18). In addition, the role of mir-340, *LGR5*, and *FOXO1* in drug resistance in several cases has also been studied (18, 19). However, the role of *miR-340* and its potential association with the *LGR5* and *FOXO1* genes as well as their effects on the drug resistance in ovarian cancer have not been addressed till now. Therefore, the aim of this survey was to evaluate and compare the correlation between the expressions of *miR-340*, *LGR5*, and *FOXO1* genes in both ovarian cancer samples and ovarian cancer cell lines (cisplatin-sensitive cell line [A2780S] and cisplatin-resistant cell line [A2780CP]) before and after the cisplatin treatment, so as to find new therapeutic procedures that do not have a negative effect on the normal fertility of ovarian cancer patients.

## 2. Materials and Methods

### Tissue preparation

In this case-control study, 30 ovarian tumor tissues samples with their peripheral tissue (as a control) were collected from Shahid Sadoughi Hospital, Iran, Yazd (November 2017 to January 2019). The exclusion criteria were: age range between 15-45 yr, addiction, long-term alcohol consumption, and family history of ovarian cancer or related malignancies. The samples were immediately put in RNAlater stabilization solution (Thermo Fisher Scientific, Waltham, MA). Next, following the histopathologic confirmation of the tumor tissues and marginal normal tissues; the collected samples were stored at a temperature of -80°C until the next initiation of steps.

### Cell culture

Besides the tissue samples in this study, both the cisplatin-sensitive (A2780S) and cisplatin-resistant (A2780CP) ovarian cancer cell lines were provided by the Iranian Institute of Pasteur Cell Bank. These cell lines were cultured according to the relevant protocols. Briefly, the cells were purchased and thawed under the laminar hood cabinet and for the first time, were grown in the medium containing 80% RPMI1640 and 20% of FBS (fetal bovine serum). In order to improve the growth rate of cells, the initial culture medium was replaced with a medium containing 90% RPMI1640 and 10% FBS after two days.

Flasks containing cell lines were then incubated at 37°C, 90% moisture, and 5% CO${}_{2}$ concentration. After the proliferation of the cells (covering the 80% of the surface of the flask), the cells were passaged and if needed, prepared for the next freeze. Next, the cells were transferred to 96-well cell culture plates for viability measurements. The IC50 was measured for cisplatin using MTT assay. Cultivate, passage, and treatment of cells was performed under conditions close to sterile and without contamination.

### MTT assay

**Figure 1:**
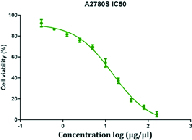
The survival rate of the cisplatin-sensitive cell line. The cisplatin-sensitive cell line was treated with different concentrations of cisplatin and IC50 was calculated. Each concentration is the average of three replicates.

MTT assays were performed as previously defined. Shortly, cells were seeded in 96-well plates (6 × 106 cell/ well) and cultured in media containing 10% FBS for 1-3 days; then, the MTT solution (5 mg/mL, 20 μL) was added into each well. After incubation for 4 hr at 37°C in a dark place, the media were taken away; 100 μL DMSO was added into each well and the plates were shaken for 30 min (in order to avoid direct contact with light and its effect on the solution color; the plate containing MTT and DMSO were wrapped in foil). The relative number of surviving cells was recorded in ELISA plate reader at 560 nm by means of evaluating the optical density (OD) of cell lysates. Finally, the cisplatin-sensitive cell line IC50 was calculated using Graph Pad Prism software (Figure 1).

### RNA extraction, cDNA synthesis, and qRT-PCR

After obtaining IC50, the cells were cultivated for 24 hr in the presence of the cisplatin in six-well plates (approximately 1 million cells/ well) to obtain a sufficient amount of cell to extract RNA. RNA was extracted from cells using the total RNA purification kit (GeneAllⓇ Hybrid-R, Cat.No. 305-101; Seoul, KOREA). The extracted RNA was then transferred and stored at a temperature of -70° C until the next stage was initiated. In the next step, the cDNA was synthesized for both *miR-340* and the *LGR5* gene according to the manufacturer's protocols provided by the BONmiR High Sensitivity MicroRNA1st Strand cDNA Synthesis Kit (Bon Yakhteh, Cat.No 0011.17.1; Tehran, Iran) and the RevertAid First Strand cDNA Synthesis Kit (Thermo Fisher Scientific, Cat.No. 4368813, 4368814, 4374966, 4374967; Waltham, MA), respectively. The synthesized cDNAs were stored at -20° C until the remaining steps were completed. Subsequently, the qRT-PCR was performed for the *miR-340* and the *LGR5* gene. The *SNORD* and *GADPH* genes were used as an internal control for the qRT-PCR reaction for *miR-340* and *LGR5* gene, respectively. The forward and reverse primers of the *LGR5*, *FOXO1*, and *GAPDH* genes were designed using the Primer3 software (Table I). The forward and reverse primers of *miR-340*, coupled cDNA synthesis kit (Cat. No. BON209001), were purchased from the Bon Yakhteh Company.

**Table 1:** Forward and reverse primers of *LGR5*, *FOXO1*, and *GAPDH* genes


**Gene**	**Forward primer**	**Reverse primer**
*LGR5*	5'- AGGATGTTGCTCAGGGTGGA-3'	5'- CTCCTCCAGGAAGCGGAGA-3'
*FOXO1*	5'- ACGAGTGGATGGTCAAGAGC-3'	5'- TCCACCAAGAACTTTTTCCAG-3'
*GAPDH*	5'-AATCCCATCACCATCTTCCA-3'	5'-TGGACTCCACGACGTACTCA-3'

### Ethical considerations

The study design was approved by the Institutional Ethics Committee of the Shahid Sadoughi University of Medical Sciences, Yazd (Code: IR.SSU.MEDICINE.REC.1396.220) and a written informed consent was obtained from each participant prior to the collection of tissue samples.

### Statistical analysis

All experiments were repeated thrice. Results are shown as mean ± SEM. All data were analyzed using the GraphPad Prism, Version 6.0. The significant differences between groups were analyzed by Student's *t* test and two-way ANOVA. P-value < 0.05 was considered statistically significant.

## 3. Results

**Figure 2:**
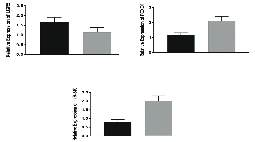
Relative expressions of *miR-340*, *LGR5*, and *FOXO1* genes in ovarian cancer tissue samples compared to the adjacent non-tumor tissues. The measurement of gene expression in tissue samples indicated a significant increase in the expression of *LGR5* gene and decreased expression of *miR-340* and *FOXO1* genes in tumor samples than in the adjacent non-tumor tissues. *GAPDH* (for *LGR5* and *FOXO1* genes) and *SNORD* (for *miR-340*) were used in order to normalize the results. Data were analyzed by paired student's *t* test and a P < 0.05 was considered as significant (Black columns represent tumor and Gray represent control specimens). *LGR5*: Leucine rich repeat containing G protein-coupled Receptor 5; *FOXO1*: Forkhead box O1; *GAPDH*: Glyceraldehyde 3-phosphate dehydrogenase; *SNORD*: Small Nucleolar RNA.

Compare to the paired adjacent normal tissues, a significant increase in the expression of the *LGR5* gene and a significant decrease in expression of the *miR-340* and *FOXO1* genes were observed in the tissue samples of the patients with ovarian cancer (Figure 2). These primary results encouraged us to continue this study. In the next step, the expression level of these genes in both cisplatin-sensitive and cisplatin-resistance cell lines (A2780S and A2780CP) was measured after 12, 24, and 72 hr of cisplatin-treatment and the results were compared to untreated cisplatin-sensitive as control. The results at this stage, in addition to confirmation of the results obtained from the measurement of gene expression in tissue samples, indicated the role of these genes in drug resistance.

**Figure 3:**
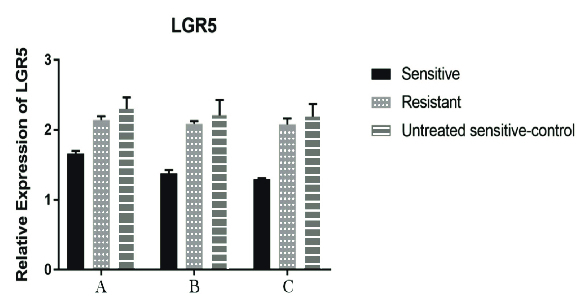
Relative expression of *LGR5* gene in the cell line groups. In cisplatin-sensitive cell lines after 24 hr of treatment, the reduction in the expression of the *LGR5* gene was clearly visible (P < 0.001). After 48 and 72 hr of treatment, the expression of the *LGR5* gene was decreased; but the expression reduction after 72 hr was not significant compared to 48 hr. In the cisplatin-resistant cell line (treated with cisplatin-sensitive IC50 cell line), a slight decrease was seen in the expression of *LGR5* gene, but this reduction was not statistically significant (P = 0.95). In each stage, the untreated cisplatin-sensitive cell line has been used as control. For both the cisplatin-sensitive and cisplatin-resistant cell lines, the IC50 of the cisplatin-sensitive cell line has been used. *GAPDH* gene was used in order to normalize the results. Data were analyzed by ANOVA and a P < 0.05 was considered significant. (Group A: 24 hr after the treatment. Group B: 48 hr after the treatment, and Group C: 72 hr after the treatment). *LGR5*: Leucine-rich repeat containing G protein-coupled Receptor 5; *GAPDH*: Glyceraldehyde 3-phosphate dehydrogenase; IC50: The half maximal inhibitory concentration.

**Figure 4:**
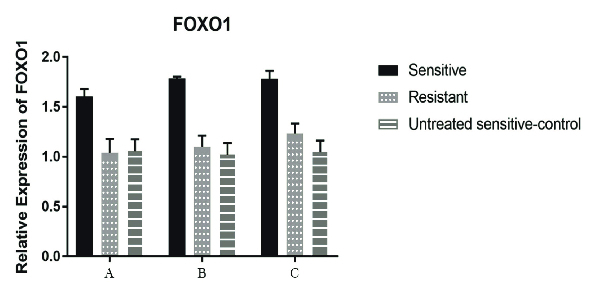
Relative expression of *FOXO1* gene in the cell line groups. In the cisplatin-sensitive cell lines after 24 hr of treatment, an increase in the expression of the *FOXO1* gene was observed (P < 0.001). Although, there was an increase in the expression of the *FOXO1* gene after 48 and 72 hr of the treatment, the increase after 72 hr was not significant compared to that after 48 hr. In the cisplatin-resistant cell line (treated with cisplatin-sensitive IC50 cell line), a slight increase in the expression of *FOXO1* gene (compared to the untreated cisplatin-sensitive at this stage) was seen after 48 hr, however, this change did not have a significant statistical value (P = 0.38). On the other hand, 72 hr after the treatment, the expression reduction was continued and at this stage, the expression reduction was significant (P = 0.01). In each stage, the untreated cisplatin-sensitive cell line has been used as the control. For both the cisplatin-sensitive and cisplatin-resistant cell lines, the IC50 of the cisplatin-sensitive cell line has been used. *GAPDH* gene was used in order to normalize the results. Data were analyzed by ANOVA test and a P < 0.05 was considered as significant. (Group A: 24 hr after the treatment; Group B: 48 hr after the treatment, and Group C: 72 hr after the treatment). *FOXO1*: Forkhead box O1; *GAPDH*: Glyceraldehyde 3-phosphate dehydrogenase; IC50: The half maximal inhibitory concentration.

**Figure 5:**
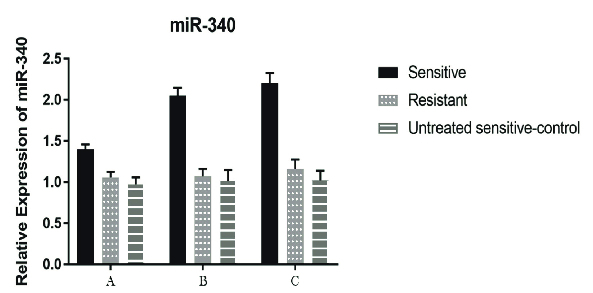
Relative expression of *miR-340* in the cell line groups. An increase in the expression level of *miR-340* gene was observed in the cisplatin-sensitive cell lines after 24 hr of treatment (P < 0.001). After 48 and 72 hr of treatment (treated with sensitive IC50 cell line), this increase in expression was more remarkable. There was no significant difference in the treatments of the cisplatin-resistant cell line. *SNORD* gene was used to normalize the results. In each stage, the untreated cisplatin-sensitive cell line was used as a control. For both the cisplatin-sensitive and cisplatin-resistant cell lines, the IC50 of the cisplatin-sensitive cell line has been used. Data were analyzed by ANOVA test and a P < 0.05 was considered as significant (Group A: 24 hr after the treatment; Group B 48 hr after the treatment, and Group C: 72 hr after the treatment). *SNORD*: Small nucleolar RNA; IC50: The half maximal inhibitory concentration.

Moreover, after 24, 48, and, 72 hr of cisplatin treatment, a significant decrease (p < 0.001) was seen in the *LGR5* expression compared to the untreated cisplatin-sensitive (control) but only in the A2780S cell line, while the A2780CP didn't show any significant alterations in the *LGR5* gene expression when compared with the untreated cisplatin-sensitive for the same periods of cisplatin treatment. Of course, the reduction of expression in the cisplatin-sensitive cell line after 48 and 72 hr of cisplatin treatment was more than that after 24 hr (Figure 3). In relation to *FOXO1* and *miR-340* genes, a significant increase in the expression after 24, 48, and, 72 hr of cisplatin treatment was seen in both genes. Although for *miR-340*, this increase was observed in all period of treatment in the A2780S cell line, the increase was very remarkable after 48 hr of cisplatin treatment (i.e., after 48 and 72 hr of cisplatin treatment), while for the *FOXO1* gene, it is noteworthy that unlike all previous treatments, a significant increase (p = 0.012) was observed after 72 hr of cisplatin treatment in the A2780CP cell line (Figures 4, 5).

## 4. Discussion

As stated below previous studies have shown that the *miR-340*, *LGR5*, and *FOXO1* genes have an effective role in the suppression or progression of various types of cancer as well as in the decrease or increase of drug sensitivity against chemotherapy.

In a study by Shi and colleagues, the function of *miR-340* and *LGR5* in breast cancer and their effect on drug resistance were discussed. As a result of this study, in addition to the introduction of the *LGR5* gene as a downstream target for the mir-340, it has also been confirmed that the *miR-340* and *LGR5* genes have a suppressive and oncogenic role in breast cancer, respectively (18). The present study confirms the results of the previous studies.

Moreover, in 2017, in a study performed by Zhang and co-workers it was shown that in patients suffering from gastric cancer, the increase in Lgr5 markers caused by the disturbance in miR-132 expression resulted in more resistant to cisplatin during the treatment process (20). The finding of this work also confirms by the outcomes of our study.

Additionally, in studies by Shi and colleagues and Song *et al* it was shown that the increased expression of *miR-340* in different pathways could be effective in increasing the sensitivity of cisplatin to hepatocellular carcinoma cell lines and osteosarcoma (21, 22). Furthermore, in recent studies, the role of *FOXO1* gene as a tumor suppressor in the EMT pathway has been discussed (23). A study by Choi and colleagues showed that theinteraction between *LGR5* and *FOXO1* genes play an important role in the development of gastric cancer (10). Finally, in previous studies, it has been shown that the interesting genes in this study can affect the fertility process through various pathways. Among these, the following are worth mentioning.

According to the previous works, a significant increase in the *LGR5* gene expression can play an important role in the infertility of women who suffer from endometriosis (11, 12). On the other hand, in some cases, disruption in the *LGR5* gene expression can induce drug resistance in a number of cancers (24). Consequently, drug resistance leads to an increased need for chemotherapy drugs prescription, which can further lead to early menopause in women that has a negative effect on their fertility (25). In addition, in the drug resistance situation, it is possible not to respond to chemotherapy treatment, in which case the patients will have to remove their ovary or ovaries. Consequently, these patients may lose their opportunity to normal fertility. Studies have also shown the role of *FOXO1* gene in infertility (26). Disturbance in the expression of the *FOXO1* gene can also affect the drug resistance induction in a number of cancers, including ovarian cancer (27). Furthermore, *miR-340* can also contribute to inducing drug resistance in cancer through the same pathways (28). As mentioned earlier, there is a significant relationship between the expressions of the *LGR5* and *FOXO1* genes (10). On the other hand, these genes are the potential downstream targets of *miR-340*. Consequently, the expression levels of the *LGR5* and *FOXO1* genes may also be affected by *miR-340*.

Inducing drug resistance can lead to late or lack of treatment in various cancers. In some cases, the patients may have to remove their ovaries. Therefore, finding new ways to reduce drug resistance can lead to easier treatment and maintaining reproductive ability in patients. The present study was designed to determine the pattern of the expressions of *miR-340*, *LGR5*, and *FOXO1* genes in ovarian cancer tissue samples as well as their association with drug resistance in ovarian cancer cell lines. As a part of this study, we were looking for pathways involved in drug resistance. Therefore, we decided to use the cisplatin-sensitive IC50 for both cisplatin-sensitive and cisplatin-resistant cell lines; we have made an attempt to find answers to these questions: (a) if a cisplatin-resistant cell line does not show a good response under IC50 of the cisplatin-sensitive cell line, what potential problem can be the cause of this issue? (b) If the cisplatin-resistant cell line shows a good response, what alterations have occurred in the expression of genes that lead to a good response?

After treating cisplatin-sensitive cell line with cisplatin at different times (24, 48, and 72 hr), we observed a significant increase in the expressions of *miR-340* and *FOXO1* genes, as well as a significant decrease in the expression of the *LGR5* gene. On the other hand, in the cisplatin-resistant cell line, significant differences in the expression of the *FOXO1* gene was detected only after 72 hr of cisplatin treatment, this may be due to the prolonged treatment of cisplatin in cisplatin-resistant cell line (up to 72 hr) and loss or reduction of resistance properties in the cisplatin-resistant cell line, given the fact that cisplatin can be toxic in a culture medium up to six days (29). Although ovarian cancer often occurs in women over the age of 45 years, this malignancy may also be observed in younger people. In this study, the tissue samples were collected from individuals aged 15-45 years, as this period is considered as the best pregnancy age in women (30), people who develop cancer during this period may lose their reproductive ability during the treatment. However, if they are treated using safe procedures in a way that the functions of their ovaries (and other organs of the reproductive system) are not affected, they will have a chance to have a natural pregnancy.

Considering the aforementioned, our hypothesis was based on the notion that the *LGR5*, *FOXO1*, and *miR-340* genes can contribute to the formation of cancer, induction of drug resistance, and infertility in women who have ovarian cancer or other gynecological malignancies. Ultimately, there is a hope that by conducting further studies, this goal can be achieved and that along with getting rid of cancer, the patients with ovarian cancer (and other related malignancies) will also have a chance to the experience natural fertility.

## 5. Conclusion

It can be concluded that the *miR-340*, *LGR5*, and *FOXO1* genes can be targeted for safer treatment and preservation of reproductive ability in patients with ovarian cancer by prevention of oophorectomy and/or high prescription of chemotherapy drugs.

##  Conflict of Interest

The authors declare that they have no conflict of interest.
